# Rationale and design of a multicenter, prospective, diagnostic clinical study: A study protocol for evaluating the diagnostic validation of deep learning-based noninvasive CT-FFR for in-stent restenosis

**DOI:** 10.1371/journal.pone.0346723

**Published:** 2026-05-06

**Authors:** Shiqi Liu, Meichen Sun, Zhao Ma, Yifei Nie, Baoen Zhang, Yuguo Xue, Mingduo Zhang, Min Zhang, Hua Chen, Xiantao Song, Dongfeng Zhang

**Affiliations:** Department of Cardiology, Beijing Anzhen Hospital, Capital Medical University, Beijing, China; Baylor Scott and White, Texas A&M College of Medicine, UNITED STATES OF AMERICA

## Abstract

**Introduction:**

Currently, there are many diagnostic strategies for in-stent restenosis (ISR) used clinically, including invasive coronary angiography (ICA), coronary computed tomography angiography (CCTA), and Fractional Flow Reserve (FFR). CCTA is not recommended for post-stent implantation patients owing to suboptimal image quality caused by artifacts. The FFR application is limited by its procedural complexity. Precise evaluation may be achieved by using computed tomography-derived fractional flow reserve (CT-FFR), which combines computational fluid dynamics (CFD) with CCTA. Anatomical and functional assessments of ISR lesions could be integrated effectively as well in this way. However, the computational complexity and prolonged processing time may hinder its utility in clinical use.

**Methods:**

This study is a multicenter, prospective, diagnostic study, aiming to establish a deep learning-based CT-FFR model for the accurate assessment of ISR and to validate its diagnostic performance using invasive FFR as the reference standard. This study will be carried out in Beijing Anzhen Hospital and 6 subcenters in China.

We planned to prospectively enroll 331 post–stent implantation patients with available CCTA data since June 2022, and invasive FFR will be performed within 3 months when clinically indicated. Patient recruitment is currently ongoing. Among them, 250 patients from Beijing Anzhen Hospital will be used to adapt and extend the existing DEEPVESSEL model, a deep learning–based CT-FFR computational software designed for the non-invasive functional assessment of coronary artery disease and previously validated in de novo coronary lesions, for application in the assessment of in-stent restenosis (ISR), and 81 patients from the other 6 subcenters will be used in external validation. Sensitivity, specificity, accuracy, positive predictive value, and negative predictive value with their corresponding 95% confidence intervals (CIs) were calculated for CT-FFR. The receiver operating characteristic (ROC) curve was analyzed, and the area under the curve (AUC) was calculated. The McNemar test and Bland-Altman plot will be used to examine the diagnostic consistency between CT-FFR and invasive FFR. The correlation was analyzed by Spearman’s correlation coefficient.

**Trial registration number:**

ChiCTR2200058822.

## Introduction

According to the 2026 Heart Disease and Stroke Statistics Update from the American Heart Association, the prevalence of coronary heart disease (CHD) among adults aged ≥20 years was 5.2% based on NHANES 2021–2023 data [1]. At present, Percutaneous coronary intervention(PCI) is still the main treatment strategy for CHD. With the increasing number of patients receiving stent implantation, the incidence of adverse events associated with stent implantation is increasing year by year. Even though drug-eluting stents (DESs) have dramatically reduced the rates of in-stent restenosis (ISR), the target lesion revascularization(TLR) caused by ISR still occurs at a rate of approximately 2% per year [2]. And compared with the PCI of de novo lesions, PCI caused by ISR is associated with a higher incidence of major adverse cardiovascular events(MACE) [3], which emphasizes the significance of early and accurate diagnosis in improving the prognosis of ISR patients.

The imaging diagnosis of ISR primarily relies on invasive coronary angiography (ICA) and coronary computed tomography angiography (CCTA). Currently, ICA remains the gold standard for ISR diagnosis, as it enables direct visualization of vascular morphology to obtain quantitative lesion parameters and allows assessment of ISR severity using the internationally standardized Mehran classification system [4]. Although CCTA demonstrates significant clinical value in evaluating acute chest pain owing to its noninvasive nature, its application in post-PCI patients remains constrained.

This stemmed primarily from imaging artifacts caused by stent material, geometric factors, and vessel wall calcification. Specifically, stent diameter, strut thickness, and associated blooming artifacts constitute major determinants of image quality degradation [5]. Additional confounding factors include patient heart rate and procedural complexity of the stent implantation, which may compromise the diagnostic accuracy of CCTA for ISR evaluation [6]. Consequently, current clinical guidelines do not recommend routine CCTA surveillance for post-PCI patients [7].

In 1993, Professor Pijls introduced the Fractional Flow Reserve (FFR). This hemodynamic index quantifies the functional significance of coronary artery stenosis by measuring the pressure ratio between the distal and proximal ends of a coronary artery stenosis [8]. Despite its diagnostic value, the widespread clinical adoption of FFR has been limited due to its invasive operation, procedural complexity, and the requirement for pharmacologically induced hyperemia.

In recent years, computed tomography-derived fractional flow reserve (CT-FFR), which combines computational fluid dynamics (CFD) with CCTA, has enabled noninvasive estimation of FFR by integrating coronary anatomical and functional data, thereby eliminating procedural risks and drug-induced adverse effects. Landmark prospective multicenter trials, including DISCOVER-FLOW [9], DeFACTO [10], and NXT [11], have consistently demonstrated the superior diagnostic performance of CT-FFR over purely anatomy-based CCTA, using invasive FFR as the reference standard. However, the computational complexity and prolonged processing time of conventional CT-FFR techniques hinder their utility in emergency settings and personalized medicine.

To overcome these limitations, researchers have proposed integrating artificial intelligence (AI) and deep learning with CT-FFR by developing deep learning algorithms trained on large-scale clinical datasets to establish fully automated CT-FFR computational models. Emerging evidence has demonstrated the clinical feasibility of this approach [12,13]. In a three-cohort randomized controlled trial, Guo et al. [13] reported that the deep learning-based automated CT-FFR system exhibited superior diagnostic performance, including higher accuracy, specificity, and area under the receiver operating characteristic curve (AUC) for detecting coronary stenosis. Notably, the system significantly reduced computational processing time and manual operator input(quantified by mouse clicks), demonstrating excellent user-friendliness.

Current research on CT-FFR has primarily focused on de novo coronary lesions, while its applicability to ISR requires further validation. In contrast to de novo lesions, which typically presents as uniform, concentric luminal narrowing due to neointimal hyperplasia, ISR has distinct diagnostic complexities. The metallic stent struts generate imaging artifacts on CCTA and alter local hemodynamics, compromising anatomical assessment accuracy. Moreover, ISR frequently manifests as diffuse lesions or multi-segment stent overlap, resulting in a poorer correlation between lumen stenosis severity and myocardial ischemia than de novo lesions [14]. Pinheiro et al. [15] conducted a comparative analysis of plaque characteristics between de novo coronary lesions and ISR lesions, demonstrating differences in neovascularization and macrophage infiltration that ultimately result in differential plaque stability and clinical outcomes.

Bozika et al. [16] systematically reviewed the clinical applications of CT-FFR, demonstrating its reliable diagnostic performance for ISR as well as its value in guiding clinical decision-making. In addition, CT-FFR enables physiology-based, precise virtual procedural planning by simulating post-intervention coronary physiology [17], thereby supporting more accurate identification of functionally relevant lesions, optimization of stent length and positioning, and reduction of potentially unnecessary revascularization, which is particularly important in patients with complex or multivessel coronary disease. Furthermore, the integration of CT-FFR with artificial intelligence has improved both diagnostic performance and computational efficiency, facilitating its implementation in routine clinical practice.

Through the development of a deep learning-based CT-FFR model incorporating optimized image reconstruction algorithms, we aim to minimize stent-induced artifacts affecting lumen boundary detection and computationally simulate the hemodynamic alterations caused by metallic stent struts, with the ultimate goal of achieving accurate diagnosis of ISR.

Therefore, we performed a multicenter, prospective diagnostic study with the following objectives: firstly, to develop a deep learning-based CT-FFR model for precise functional assessment of ISR, and secondly, to validate its diagnostic performance using invasive FFR as the reference standard.

## Method

### Study design

**Fig 1:**
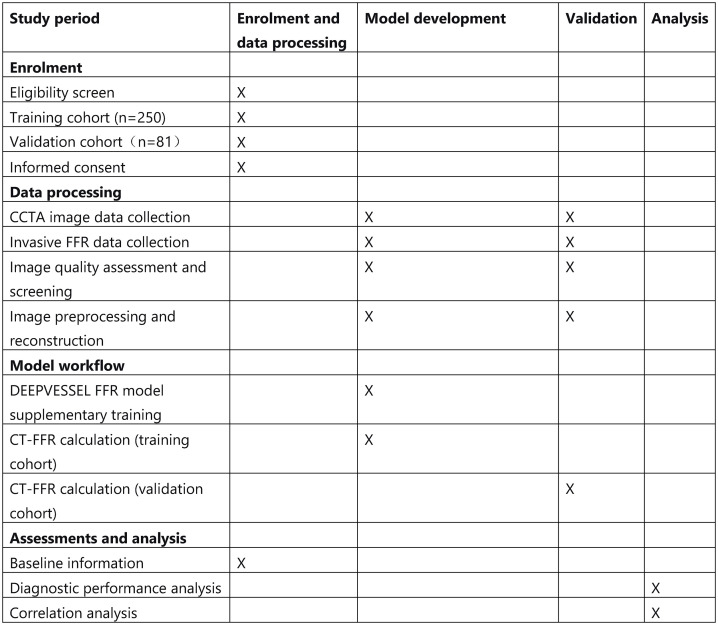
Schedule of the trial.

Adhering to the Standard Protocol Items: Recommendations for Interventional Trials (SPIRIT) 2013 Statement, we have outlined the trial design and protocol in detail (Fig 1).

**Fig 2:**
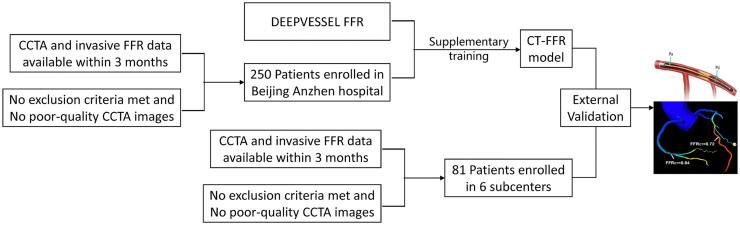
Study diagram.

This study will be carried out in Beijing Anzhen Hospital and 6 subcenters (Fuwai Hospital, Chinese Academy of Medical Sciences, Beijing Tongren Hospital, Beijing Hospital, Beijing Chaoyang Hospital, Xuanwu Hospital) in China. From June 2022, consecutive post-stent patients with available CCTA data were prospectively enrolled. Invasive FFR will be performed according to clinical and imaging indications within 3 months of CCTA. For the purposes of supplementary training and diagnostic performance analysis, only patients with available invasive FFR measurements will be included. Patients without invasive FFR data will be excluded. Among them, 250 patients from Beijing Anzhen Hospital will be used to adapt and extend the existing DEEPVESSEL model [18], a deep learning–based CT-FFR computational software designed for the non-invasive functional assessment of coronary artery disease and previously validated in de novo coronary lesions, for application in the assessment of in-stent restenosis (ISR), and 81 patients from the other 6 subcenters will be used in external validation (Fig 2).

The main inclusion criteria were post-stent patients with available CCTA data, and invasive FFR was performed within 3 months according to clinical and imaging indications. The main exclusion criteria are: (1)recent(＜3 months) acute coronary syndrome, (2)coronary artery bypass grafting(CABG) history, (3)severe calcification(Agatston≥400), (4) left ventricular ejection fraction(LVEF)≤40%, (5)significant valvulopathy, (6)cardiac arrhythmias requiring therapeutic intervention, or (7)cardiac metallic devices(e.g., prosthetic valves, cardiovascular implantable electronic devices). (Table 1)

**Table 1:** Inclusion and exclusion criteria.

Inclusion criteria	Exclusion criteria
(1) post-stent patients with available CCTA data	(1) recent (<3 months) acute coronary syndrome
(2) invasive FFR was performed within 3 months according to clinical and imaging indications	(2) coronary artery bypass grafting (CABG) history
	(3) severe calcification(Agatston≥400)
	(4) left ventricular ejection fraction(LVEF)≤40%
	(5) significant valvulopathy
	(6) cardiac arrhythmias requiring therapeutic intervention
	(7) cardiac metallic devices (e.g., prosthetic valves, cardiovascular implantable electronic devices)

This study is a prospective study, and informed consent has been obtained. This trial does not involve collecting biological samples for storage.

### Study process

Prospective patients enrollment for this study began in June 2022 and is currently ongoing. Data collection (including demographic information, imaging data, laboratory tests, and interventional procedure parameters) and data analysis are scheduled to be finalized in 2026.

During the data collection process, all information will undergo rigorous de-identification to ensure that researchers will not have access to any personally identifiable information of participants at any stage.

Patients will be prospectively enrolled into the study, and their CCTA and invasive FFR data will be systematically collected according to this protocol. All data, including demographic characteristics, medical history, laboratory results, and clinical examinations (ECG, ICA, CCTA, and echocardiography), will be systematically recorded and stored using an electronic data capture (EDC) system.

All CCTA examinations will be acquired using multidetector CT scanners with ≥64 detector rows, in strict adherence to the 2022 Society of Cardiovascular Computed Tomography(SCCT) guidelines. When the CCTA image quality is suboptimal (e.g., obvious motion artifacts or blooming artifacts) or couldn’t satisfy the subsequent CT-FFR analysis, the patient will be censored out. Because this study is still ongoing and patient enrollment has not yet been completed, patients excluded due to poor image quality will be replaced by continued recruitment until the predefined sample size is reached.

FFR will be performed in intermediate stenosis (30–90% stenosis in vessel diameter) with a vessel size of at least 2 mm based on clinical symptoms suggestive of myocardial ischemia according to standard practice by experienced invasive cardiologists. Pressure calibration should be confirmed as zero at the proximal segment of the coronary arteries. After calibration, the pressure wire should be advanced to the distal end of the stenosis. Maximal myocardial hyperaemia should be induced by continuous intravenous adenosine infusion via a central or peripheral vein with an infusion rate of 140 μg/kg/min. At the same time, the distal coronary artery pressure at the pressure sensor and the proximal pressure at the coronary ostium will be recorded. A pullback recording should be performed and recorded. The absence of pressure signal drift needs to be confirmed at the distal end of the guiding catheter.

To ensure consistency across centers, the same criteria for FFR indication and standardized procedural protocols had been applied at Beijing Anzhen Hospital and all participating subcenters. All investigators underwent consistent training before study initiation.

CCTA and invasive FFR data of 250 prospectively enrolled patients at Beijing Anzhen Hospital will be transmitted to Keya Medical for supplementary training of the existing DEEPVESSEL FFR model. Following model establishment, the algorithm will be applied to analyze CCTA images from 81 prospectively enrolled patients in the 6 subcenters to derive CT-FFR values for external validation. Diagnostic performance for ISR detection will be rigorously evaluated using invasive FFR as the reference standard (threshold ≤0.80).

### CT-FFR calculation

We will employ a Deep Bidirectional Long-term Recurrent Neural Network (DBL-RNN) algorithm, independently developed by Keya Medical, which integrates a Multi-Layer Perceptron (MLP) and a Bidirectional Recurrent Neural Network (Bi-RNN). The framework will consist of two core components: CCTA image processing and deep learning–based CT-FFR calculation.

In the CCTA image processing and feature extraction stage, a Multi-Layer Perceptron (MLP) will be utilized for fully automated three-dimensional coronary artery reconstruction, including centerline extraction and lumen segmentation. Based on the reconstructed coronary geometry, a series of morphological and hemodynamics-related local feature vectors will be extracted point-by-point along the vessel centerline. These feature vectors include local vascular features (e.g., coronary cross-sectional area, coronary radius, distance to the nearest upstream coronary bifurcation), local stenotic features (e.g., stenosis length and the smallest 50% coronary radius along the stenosis), and global features (including upstream and downstream vascular characteristics and stenosis features).

The deep learning component adopts a Bidirectional Recurrent Neural Network (Bi-RNN) architecture. The sequential feature vectors generated from the previous step will be input into the Bi-RNN, which enables bidirectional information propagation and simultaneously accounts for the local upstream and downstream context of each centerline point as well as its global relationship within the entire coronary tree. Through this architecture, the network will infer continuous CT-FFR values along the entire coronary artery tree.

The DBL-RNN algorithm will be trained using data from 250 patients prospectively enrolled at Beijing Anzhen Hospital. Model weights will be initialized using a calibrated normal distribution to stabilize gradients during the early training phase. To mitigate overfitting, dropout regularization with a rate of 0.5 will be applied to the hidden layers of the network. The loss function will be defined as the squared error (SE) between the CT-FFR values generated by the model and the invasive FFR measurements. Then, the network will be iteratively optimized to minimize this discrepancy, thereby driving the predicted CT-FFR values toward the invasive reference standard. Model optimization will be performed using the Adam optimizer, with a learning rate of 0.0001 and exponential decay rates for the first- and second-moment estimates (β1 = 0.9, β2 = 0.999).

Within the training cohort, internal validation will be performed using a five-fold cross-validation approach. The dataset will be randomly divided into five approximately equal folds. In each iteration, four folds will be used for model training, and the remaining fold will be used for validation. This procedure will be repeated five times, with each fold serving as the validation set once.

Upon completion of training, the model could generate continuous CT-FFR values along the entire length of each coronary artery. All coronary vessels will be labeled according to the 18-segment classification system of the Society of Cardiovascular Computed Tomography (SCCT). During the validation phase, invasive coronary angiography images acquired at the time of invasive FFR measurement will be carefully reviewed to identify the exact anatomical location of the pressure-wire sensor within the three-dimensional coronary model. Then, the corresponding CT-FFR value at this location will be extracted, ensuring precise spatial matching between CT-FFR and invasive FFR measurements.

During the model training phase, CCTA images from the 250 patients enrolled at Beijing Anzhen Hospital will be paired with their corresponding invasive FFR measurements and used as input–output data for model training and parameter optimization.

During the model validation phase, data from 81 patients enrolled at five additional subcenters will be analyzed under a strictly blinded study design. Investigators responsible for CT-FFR computation will have access only to anonymized CCTA images and will be fully blinded to invasive FFR results, coronary angiography reports, and all clinical diagnostic information. Conversely, cardiologists interpreting invasive FFR measurements will be blinded to the CT-FFR results and will base their assessments solely on the original angiographic images and pressure recordings.

After CT-FFR values and invasive FFR measurements for the validation cohort are independently generated, a third-party investigator, who is not involved in any prior analytical steps, will match the two datasets according to predefined anonymized identification numbers. All diagnostic performance analyses will be subsequently performed by statisticians who have access only to the paired numerical values and no access to the original imaging data or clinical reports.

### Statistics

All statistical analyses will be performed using Stata version 17.0. The categorical data will be presented as counts with percentages and analyzed using the chi-squared test. Normally distributed continuous variables will be presented as the means ± standard deviations and analyzed using independent sample t-tests. Non-normally distributed continuous variables will be presented as the median (interquartile range) and analyzed using the Mann–Whitney U test. All tests will be two-tailed with p < 0.05 significant.

Baseline demographic, clinical characteristics, and angiographic characteristics, including the percentage of diameter stenosis, will be prospectively collected. These variables will be separately described in the training cohort and the external validation cohort. Comparisons between these two cohorts will be performed to assess comparability, thereby facilitating appropriate interpretation of external validation results. Because FFR primarily performed in intermediate stenosis lesions (30–90% stenosis in vessel diameter), the proportion of intermediate (30–90%) diameter stenosis lesions will be summarized and compared between the two cohorts.

Using invasive FFR as the reference standard, the diagnostic performance of CT-FFR will be evaluated at both the vessel level and the patient level. Only stented segments that undergo invasive FFR measurement will be included in the diagnostic performance analysis. Stented segments without available invasive FFR measurements will not be included in the statistical evaluation due to the absence of a reference standard.

Diagnostic performance of CT-FFR will be evaluated using invasive FFR as the reference standard. A threshold of 0.80 will be used for both CT-FFR and invasive FFR to define functionally significant lesions. Sensitivity, specificity, positive predictive value, negative predictive value, and overall accuracy will be calculated. Receiver operating characteristic (ROC) curves will be constructed, and the area under the curve (AUC) will be calculated to assess diagnostic discrimination.

At the vessel level, because a single patient may contribute more than one stented vessel, vessel-level observations may not be independent. Therefore, diagnostic performance metrics and their 95% confidence intervals will be estimated using a cluster bootstrap resampling procedure at the patient level (1000 iterations), in which patients will be sampled with replacement, and all corresponding vessel-level observations will be retained in each bootstrap sample.

Agreement between CT-FFR and invasive FFR classifications will be assessed using a cluster-adjusted McNemar test implemented with generalized estimating equations (GEE) with an exchangeable correlation structure to account for within-patient clustering. Continuous CT-FFR and invasive FFR measurements will be assessed using Spearman correlation analysis. Agreement between the two measurements will be further evaluated using Bland–Altman analysis with a linear mixed-effects model, with patient identification number included as a random effect to account for clustering. The mean bias and 95% limits of agreement will be derived from the mixed-effects model.

At the patient level, when multiple stented vessels were present, the vessel with the lowest invasive FFR value will be selected to represent that patient.

The results from the above statistical analyses will be presented in the final study report.

### Sample size calculation

The sample size calculation was based on previous studies [19] and the following hypothesis: At the patient level, CT-FFR demonstrated an AUC of 0.73(95% CI: 0.55–0.87) for detecting ISR, with a disease prevalence of 76%. A sample size calculation was performed using PASS 2021 (Test for One ROC Curve) with α = 0.05, power = 0.9, and two-tailed testing, yielding a required sample size of 77 patients. We planned to prospectively enroll 81 patients in the external validation cohort, which was expected to provide 90% power to detect an AUC of 0.73.

The training cohort size was defined based on data availability and a predefined training-to-validation ratio of approximately 3:1 to balance model development and independent performance evaluation. Therefore, at least 243 patients were required for model training. Considering clinical practice and data availability, we prospectively planned to enroll 250 patients to meet the sample size requirement, which is comparable to or even larger than the training cohort sizes reported in previous study [20].

### Ethics

The study protocol was approved by the Ethics Committee of Beijing Anzhen Hospital. (Approval No: KS2022005; Date of approval: February 11, 2022).

### Study management and conduct

A Trial Steering Committee (TSC), including clinical doctors from Beijing Anzhen Hospital and engineers from Keya Medical, oversees protocol approval, major decisions, and membership adjustments. A Data Management Team maintains the Electronic Data Capture (EDC) system, electronic Case Report Forms(eCRFs), Electronic Medical Records (EMRs), and real-time quality control.

The primary results and initial manuscript will be developed in the name of this study by the writing committee, which was approved by the steering committee. The writing committee will be composed of members of the steering committee, statisticians, and researchers. They will compile major reports in the name of this study. Results of this study will be published and presented in a variety of formats, including presentations at relevant medical conferences and published in academic journals.

All protocol amendments must receive prior approval from the principal investigator, regulators (when applicable), and the institutional ethics committee. However, protocol amendments may be implemented before ethics approval when necessary to ensure subject safety. While formal approval processes are required for any amendments, investigators may take immediate emergency measures to protect participant welfare, even if these actions deviate from the original protocol. In such cases, the institutional review board/ethics committee (IRB/EC) must be informed promptly and formally.

### Patients and public involvement statement

There was no patient or public involvement in the design of the present study.

## Discussion

This study is a multicenter, prospective, diagnostic study, aiming to establish a deep learning-based CT-FFR model for the accurate assessment of ISR and to validate its diagnostic performance using invasive FFR as the reference standard. The expected outcome is a strong correlation and good diagnostic consistency between the CT-FFR model and invasive FFR. Through the development of this model, the anatomical and functional assessments of ISR lesions are effectively integrated, reducing the impact of stent structure and other factors on CCTA image quality. This model enables precise diagnostic results without invasive procedures, significantly optimizing the diagnostic process and improving treatment efficiency.

Previous studies on CT-FFR have primarily focused on de novo coronary lesions, with limited research on the CT-FFR model for ISR lesions. Therefore, this study specifically constructs a CT-FFR model for ISR lesions. Compared to other studies, this study was based on a fully automated CCTA image processing and deep learning–based analytical framework using a Deep Bidirectional Long-term Recurrent Neural Network (DBL-RNN) for CT-FFR estimation. The framework performs automated three-dimensional reconstruction of the coronary arteries and centerline-level feature extraction. Local vascular morphology, stenosis-related characteristics, and upstream and downstream global information are integrated within a bidirectional recurrent neural network, which captures relationships along the vessel path and the overall structure of the coronary artery tree. As a result, continuous CT-FFR values can be estimated along the entire coronary artery. During model training, dropout regularization and Adam optimizer were applied to improve model stability and generalizability, providing a novel approach for non-invasive functional assessment of coronary lesions.

Multiple clinical trials have demonstrated that despite the widespread application of CCTA, a considerable proportion of patients still undergo unnecessary ICA following CCTA. Furthermore, factors such as stent artifacts and coronary artery calcification further reduce the diagnostic accuracy of CCTA for ISR lesions. While ICA can precisely demonstrate the severity of coronary stenosis, substantial evidence indicates the discordance between anatomical and functional assessments of stenosis severity. Moreover, compared with CCTA, direct ICA in patients with stable CAD is associated with higher risks of MACE and procedure-related complications [21,22]. Invasive FFR is difficult to be widely used in clinical practice due to its invasive procedure and technical complexity.

Tsigkas et al. [23] systematically reviewed a range of techniques for both functional and anatomical assessment of coronary artery disease, including CT-FFR. Although these approaches do not require the use of hyperemic agents such as adenosine, CT-FFR avoids invasive coronary angiography compared with angiography-based functional assessments (e.g., QFR), and does not rely on pressure-wire–based measurements when compared with wire-based physiological indices (e.g., iFR and RFR). Consequently, CT-FFR provides a faster and less invasive assessment strategy, which may support wider clinical use and improve the efficiency of clinical decision-making. These advantages are particularly relevant in patients with prior stent implantation, in whom repeated invasive assessment may associated with higher procedural risk.

Although CT-FFR addresses these limitations and has shown good diagnostic performance in studies [24], its clinical utility remains constrained by massive computational demands and a time-consuming process, making it difficult to implement for individualized patient care, particularly in emergency scenarios.

To address these challenges, we used artificial intelligence and machine learning techniques to analyze a large amount of clinical data of post-stent implantation patients, including CCTA, ICA, and FFR, and successfully established the CT-FFR model. The development of this model significantly reduces CT-FFR computation time while maintaining high diagnostic accuracy and specificity, demonstrating broad potential for clinical application.

The ACCURATE-CT clinical trial has established that AccuFFRct, a machine learning-derived noninvasive FFR model, demonstrates effective risk stratification and high diagnostic accuracy for detecting myocardial ischemia in patients with coronary artery disease. Importantly, this technology shows consistent performance across various clinical presentations, stenosis severity levels, anatomical locations, and “gray zone” cases [25]. Other studies have further validated that deep learning-based CT-FFR models are associated with reduced ICA referral rates and lower MACE incidence, demonstrating their clinical value in guiding therapeutic decision-making and optimizing ICA utilization efficiency [26–28].

## Limitation

First, patients with coronary artery bypass grafting (CABG), acute coronary syndrome (ACS), and other specific conditions were excluded. In addition, although this was a multicenter study, both the supplementary training and external validation cohorts were derived from Beijing Anzhen Hospital and six other hospitals located in Beijing. Therefore, the applicability of the model and long-term prognostic value for post-stent implantation patients requires further investigation.

Second, an internal validation of coronary artery segmentation accuracy was not planned in this study. Although the segmentation component is based on an established and previously validated CCTA processing framework, segmentation accuracy was not evaluated as a primary endpoint in the present study, which focused primarily on the diagnostic performance of CT-FFR. Consequently, residual segmentation errors may have influenced CT-FFR estimation in certain cases. Future studies incorporating systematic assessment of segmentation accuracy and its impact on CT-FFR performance may further enhance model interpretability and robustness.

In addition, exclusion of patients with suboptimal CCTA image quality may introduce selection bias. Patients with severe coronary calcification or substantial imaging artifacts may be excluded from the final analysis cohort. Consequently, the diagnostic performance of the trained model may be overestimated and may not fully reflect its performance in real-world populations with suboptimal imaging conditions.

Finally, invasive FFR is performed according to clinical and imaging indications rather than as part of a study-mandated protocol uniformly applied to all patients. Consequently, referral and verification bias may be present. If a relatively high prevalence of functionally significant ISR is observed in final results, this would suggest that the cohort represents a clinically selected population rather than a real-world post-stent population. As a result, the reported diagnostic performance, including sensitivity, specificity, and AUC, may not fully reflect performance in broader post-stent populations. Further validation in less selected populations will be warranted.

## Supporting information

S1 File SPIRIT-checklist. (DOC)

S2 File Study protocol in Chinese. (DOCX)

S3 File Study protocol in English. (DOCX)

S4 File Ethics approval in English. (DOCX)

S5 File Copy of original Ethics approval. (PDF)

S6 File Informed consent of the first patient. (PDF)

S7 File Informed consent of the first patient in English. (DOCX)
