# Supplementary material for: Rationale and design of a multicenter, prospective, diagnostic clinical study: A study protocol for evaluating the diagnostic validation of deep learning-based noninvasive CT-FFR for in-stent restenosis
Source: PLoS One. 2026 May 6;21(5):e0346723. doi: 10.1371/journal.pone.0346723 (PMC13148680; doi:10.1371/journal.pone.0346723)
Supplement: S2 File — (DOCX) [file pone.0346723.s002.docx]

**基于深度学习的无创血流储备分数（CT-FFR）评估支架内再狭窄的研究方案**

版本号：V1.0

版本日期：2021年12月08日

研究专用-机密

不得用于本研究以外的任何用途

本项目方案及子课题研究方案由研究指导委员会制订，该委员会对其内容拥有知识产权。未经研究项目组事先批准，而因本研究以外的任何目的复制或使用本方案中的信息和数据，均属侵权。

**主要研究者声明**

我已阅读以下方案：

基于深度学习的无创血流储备分数（CT-FFR）评估支架内再狭窄的研究

版本和日期：2021年12月08日，1.0版

我已经阅读了该研究方案，并同意它包含进行本研究的所有必要细节。我将按照本文所述进行研究，并在计划的时间内完成本研究。

我将向所有参与本研究的研究人员提供研究方案和所有相关信息的副本。我将与他们讨论这些材料，以确保他们充分了解研究干预措施和研究组织实施情况。

| **主要研究者**  张东凤教授  国家心血管疾病临床医学研究中心  首都医科大学附属北京安贞医院 | *日期* | *签名* |
| --- | --- | --- |

1. **简介****（背景和理论基础）**

随着社会经济迅猛发展，国民生活状态发生了深刻的变化，包括人口老龄化及心血管病危险因素的增加，导致冠心病发病率逐年上升。经皮冠状动脉介入治疗（percutaneous coronary intervention, PCI）是冠心病患者的主要治疗方式之一。注册数据显示，2009-2019年中国大陆地区PCI治疗例数逐年上涨，2020年尽管受到疫情影响，大陆地区冠心病介入治疗的总病例数仍高达近100万例。

支架内再狭窄（in-stent restenosis，ISR）定义为通过冠状动脉造影确定的支架节段或其边缘（距离支架边缘5mm内的节段）出现超过50%参考血管直径的狭窄 [1]。金属裸支架时代ISR发生率高达20-40%。尽管支架构造不断改良，在药物洗脱支架时代，ISR发生率仍高达5%-10% [2]，而ISR与主要不良心血管事件密切相关 [3]。鉴于基数庞大且持续扩张的介入治疗现状，ISR必然是目前甚至未来将要面临的重大难题。

有创冠状动脉造影（invasive coronary angiography，ICA）是诊断ISR的金标准，但其为有创性检查，患者依从性差，而且造影本身存在麻醉剂过敏、导丝打结、穿刺血管闭塞以及穿刺部位出血、血肿、动静脉瘘等多种并发症可能，因此不适宜作为临床筛查ISR的首选方案。冠状动脉计算机断层扫描血管造影（coronary computed tomography angiography，CCTA）是目前实际诊疗工作中应用较为普遍的筛查方法 [4-7]。但是，CCTA对ISR的诊断准确性受支架的直径、厚度、材质以及血管钙化程度的影响，ISR病变部位也会对CCTA诊断准确性产生影响。

临床上可通过评估血管狭窄远段的血流储备分数（fractional flow reserve，FFR）数值来评价由狭窄引起的血流动力学改变，从而判断是否存在心肌缺血以及心肌缺血的严重程度，最终使患者最大程度获益并避免过度治疗。计算流体动力学（computational fluid dynamics，CFD）是计算机技术和流体力学结合的交叉学科，CFD技术已应用于血管里血液流动、气管里气体/颗粒流动仿真模拟等场景，其中将CFD和CCTA结合可以推导冠状动脉内血流的流体动力学参数，从而计算出整个冠脉树上的FFR值。近年来，这种基于CCTA技术发展的FFR评估技术已成为冠状动脉病变无创功能学评价的一种有效方法。

我国自主研发的DEEPVESSEL FFR产品集合了人工智能、医学影像、生物医学工程等相关学科的关键技术，采用自主研发的基于序列结构的深度学习技术对人体循环系统血管疾病进行智能精准评估，快速、有效、无创地计算用于评估心肌缺血程度的FFR，实现精确高效的检测，并达到低成本诊断的目的。

针对目前冠心病介入治疗术后ISR的筛查难题，建立基于人工智能的精准评估ISR的CT-FFR模型，明确新模型的诊断准确性及指导治疗策略的可行性。通过精准、无创、高效评估ISR，给予个体化精准治疗，避免过度诊疗和误诊漏诊，改善植入支架人群的预后，提升北京市乃至全国的冠心病诊疗能力，同时助力优质医疗资源下沉，推动分级诊疗。

参考文献：

[1] Motoyama S, Ito H, Sarai M, et al. Ultra-High-Resolution Computed Tomography Angiography for Assessment of Coronary Artery Stenosis. Circ J. 2018;82(7):1844-1851.

[2] Ajmal M, Chatterjee A, Acharya D. Persistent or Recurrent Angina Following Percutaneous Coronary Revascularization. Curr Cardiol Rep. 2022;24(12):1837-1848.

[3] Alfonso F, Pérez-Vizcayno MJ, Cárdenas A, et al. A Prospective Randomized Trial of Drug-Eluting Balloons Versus Everolimus-Eluting Stents in Patients With In-Stent Restenosis of Drug-Eluting Stents: The RIBS IV Randomized Clinical Trial. J Am Coll Cardiol. 2015;66(1):23-33.

[4] 林祥腾,纪凤颖,张琳焓,常燃.256层极速CT对冠状动脉不同类型支架通畅性评价[J].医学综述,2013,19(4):736-739

[5] Rixe J, Achenbach S, Ropers D, et al. Assessment of coronary artery stent restenosis by 64-slice multi-detector computed tomography. Eur Heart J. 2006;27(21):2567-2572.

[6] 刘健萍,高明勇,刘辉,刘再毅,梁长虹.支架材质对冠状动脉支架CTA成像影响[J].医学影像学杂志,2016,26(7):1208-1211

[7] Maintz D, Burg MC, Seifarth H, et al. Update on multidetector coronary CT angiography of coronary stents: in vitro evaluation of 29 different stent types with dual-source CT. Eur Radiol. 2009;19(1):42-49.

1. **管理信息**

**2.1试验/研究注册**

研究方案已经过首都医科大学附属北京安贞医院伦理委员会和其他分中心伦理委员会审核并批准，注册号为KS2022005，并在中国临床试验注册中心进行注册，注册号为ChiCTR2200058822。

**2.2研究支持单位**

科亚医疗为本研究协作单位。主要职责为数据分析，并配合承担单位进行项目结果解释，协助进行科研论文撰写、专利申请等。

**2.3研究人员与职责**

**2.3.1**

主中心：首都医科大学附属北京安贞医院

主要研究者：宋现涛、张东凤、马钊、孙美辰、聂一飞、刘世淇

分中心：首都医科大学宣武医院

主要研究者：李静、肖克令

分中心：北京医院

主要研究者：于雪、李彭

分中心：首都医科大学附属北京同仁医院

主要研究者：郭彩霞、江雪

分中心：首都医科大学附属北京朝阳医院

主要研究者：徐立、李闯

分中心：中国医学科学院阜外医院

主要研究者：钱杰

**2.3.2**

联系信息：张东凤，dongfengdoctor@outlook.com

**2.3.3**

本研究是一项由研究者发起的临床研究，本研究重要的科学决策和研究管理由指导委员会完成。

**2.3.4**

由申办方或申办方委托的合同制临床研究管理机构负责本研究的项目管理和协调运营。其职责包括但不限于：项目和数据管理、与本研究各委员会成员协调、协助研究者进行伦理委员会申报和进度报告、协助撰写完善研究方案、培训本研究参与单位的人员、研究中心启动、监查和稽查、监控数据质量和数据安全、监控研究方案、医学指南、法律法规的遵守和执行情况、协助和组织准备研究数据和文章发表的相关事宜。

**项目成立指导委员会**（Steering Committee，SC），由主中心及分中心的专家组成，负责批准最终的研究方案，并在临床研究进行、结果分析和报告过程中对重要问题进行决策。指导委员会有权增加新的成员，以加强研究执行和研究分析的完整性。指导委员会成员还包括科亚医疗相关成员。

**数据管理团队**：负责维护研究的IT系统和数据输入、数据验证。本研究将使用电子数据采集（Electronic Data Capture，EDC）系统进行数据收集和管理。数据管理团队将设计电子CRF表，设计及维护电子病历，并在项目实施过程中对数据质量进行监控。

**2.4缩略词和术语表**

| CI | 置信区间 |
| --- | --- |
| SD | 标准差 |
| CRF/eCRF | 病例报告表/电子病例报告表 |
| DSMB | 数据和安全监察委员会 |
| EC | 伦理委员会 |
| EDC | 电子数据采集系统 |
| GCP | 临床试验质量管理规范 |
| CRO | 合同研究组织 |
| IC | 知情同意 |
| ICMJE | 国际医学期刊编辑委员会 |
| PI | 主要研究者 |
| SC | 指导委员会 |

**2.5研究方案概要**

| 标题 | 基于深度学习的无创血流储备分数（CT-FFR）评估支架内再狭窄的研究 |
| --- | --- |
| 研究目的 | 建立基于人工智能的精准评估ISR的CT-FFR模型，明确新模型的诊断准确性及指导治疗策略的可行性 |
| 研究设计 | 多中心、前瞻性、诊断性临床研究 |
| 研究金标准 | 有创FFR |
| 研究人数 | 331人 |
| 研究持续时间 | 48个月 |
| 研究内容 | 以模型补充训练后，使用有创FFR作为金标准，得到冠脉支架术后CT-FFR模型的诊断效能，并在外部进行验证。 |
| 入选标准 | 患者必须满足以下所有条件才能纳入研究：  （1）冠状动脉支架术后患者；  （2）冠状动脉造影术前3月内完善冠状动脉CTA检查。 |
| 排除标准 | 患者如果符合排除标准的任何一项，将不能纳入研究：  （1）既往3月内存在急性冠脉综合征  （2）既往冠脉搭桥  （3）冠脉重度钙化（CACS ≥ 400）；  （4）心力衰竭、严重瓣膜疾病、严重的需干预的心律失常、既往其他心脏金属植入物（如机械瓣、起搏器等）；  （5）其它原因研究者认为不适合入选的受试者。 |
| 剔除标准 | 冠状动脉CTA图像质量欠佳无法满足后续CT-FFR分析 |
| 统计方法 | 使用有创FFR作为金标准，采用Spearman相关系数和Bland-Altman分析法分析冠脉支架术后CT-FFR模型与QCA的相关性；计算冠状动脉支架术后CT-FFR模型的敏感性、特异性、准确性、阳性预测值和阴性预测值并计算受试者工作特征曲线（ROC）分析的曲线下面积（AUC），并在内部及外部进行模型验证，评估训练得到的冠状动脉支架术后CT-FFR模型效果。双尾P值<0.05被认为具有统计学意义。 |
| 数据管理 | 研究将使用电子CRF进行数据收集和管理 |

**3．研究内容**

**3.1 研究目的**

建立基于人工智能的精准评估ISR的CT-FFR模型，明确新模型的诊断准确性及指导治疗策略的可行性。

**3.2研究设计**

本研究为多中心、前瞻性、诊断性临床研究。

本研究计划在首都医科大学附属北京安贞医院和其他分中心（中国医学科学院阜外医院、首都医科大学附属北京同仁医院、北京医院、首都医科大学附属北京朝阳医院、首都医科大学宣武医院）于2022.6开始前瞻性纳入进行了冠脉造影且存在可获取的3个月内CCTA和有创FFR资料的支架植入术后患者331人。其中在本中心纳入的250人用于对现有 DEEPVESSEL FFR 模型的补充训练，分中心纳入的81人用于准确性验证。

**4．研究方法**

**4.1研究中心选择**

本研究由首都医科大学附属北京安贞医院发起和主导，前瞻性患者纳入和数据收集将于2022年6月开始，计划在首都医科大学附属北京安贞医院、中国医学科学院阜外医院、首都医科大学附属北京同仁医院、北京医院、首都医科大学附属北京朝阳医院、首都医科大学宣武医院前瞻性收集符合纳排标准的病人。

**4.2纳入及排除标准**

4.2.1纳入标准

患者必须满足以下所有条件才能纳入研究：

（1）冠状动脉支架术后患者；

（2）冠状动脉造影术前3月内完善冠状动脉CTA检查。

4.2.2排除标准

患者如果符合排除标准的任何一项，将不能纳入研究：

（1）既往3月内存在急性冠脉综合征

（2）既往冠脉搭桥

（3）冠脉重度钙化（CACS ≥ 400）；

（4）心力衰竭、严重瓣膜疾病、严重的需干预的心律失常、既往其他心脏金属植入物（如机械瓣、起搏器等）；

（5）其它原因研究者认为不适合入选的受试者。

4.2.3剔除标准

冠状动脉CTA图像质量欠佳无法满足后续CT-FFR分析

**4.3 CT-FFR建模技术过程**

我们使用科亚医疗自主研发的的深层双向长期递归神经网络（Deep Bidirectional Long-term Recurrent Neural Network，DBL-RNN）算法，该算法融合了多层感知神经网络和双向递归神经网络。主要分为CCTA图像处理和深度学习两个核心部分。

在CCTA图像处理与特征提取部分，首先，利用多层感知神经网络对原始CCTA图像进行全自动化的冠状动脉三维重建，包括中心线提取和管腔分割。在此基础上，沿血管中心线逐点提取一系列形态学与血流动力学相关的局部特征向量。向量包括：局部血管特征（横截面积、冠脉半径、到最近上游冠脉分叉点距离等）、局部狭窄特征（狭窄长度和the smallest 50% coronary radius along stenosis）和全局特征（上下游血管特征和狭窄特征）。

深度学习部分采用双向递归神经网络（Bi-RNN）架构。将上述步骤生成的特征向量序列输入Bi-RNN，该网络通过双向信息传递，同时考虑每个中心线点的局部上下游及其在整个血管树中的全局关系，从而精准推断整个冠状动脉树上连续的CT-FFR值。

**4.4样本量计算**

样本量的计算基于之前的研究和以下假设：在病人层面，CT-FFR对于支架内再狭窄的AUC为0.73（0.55-0.87），患病率为0.76。使用PASS 2021的Test for One ROC Curve功能计算，设置α=0.05，power=0.9，双侧检验，得到样本量分别为77例。我们计划前瞻性纳入81例患者至外部验证队列，预计能以90%的检验效能检测出AUC为0.73的效果。

训练队列的规模基于数据可及性及预设的训练集与验证集比例（约为3:1）确定，以平衡模型开发与独立性能评估的需求。因此，模型训练至少需要243例患者。结合临床实践与数据可及性，我们前瞻性计划纳入250例患者以满足样本量要求，该规模与先前研究报道的训练队列规模相当或更大。

**5.数据收集、管理和分析**

**5.1数据收集内容**

5.1.1人口学资料

年龄、性别、身高、体重、住院及出院日期。

5.1.2病史信息

患者入院的主诉、现病史、既往史（高血压、糖尿病、血脂异常、陈旧性心梗及病程），个人史（吸烟、饮酒），用药史（患者既往用药情况），家族史（心血管相关疾病）、既往PCI时间。

5.1.3实验室检查

血常规、CRP、hs-TnI/TNI、CK-MB、BNP、NT-pro BNP、D-Dimer、FDP、肝肾功能、血脂、血糖等结果。

5.1.4临床检查

生命体征和体格检查：记录患者入院时的血压、心率；

心电图：记录患者入院时心电图；

冠脉CTA：记录患者入院时冠脉CTA相关内容（包括检查时间、钙化积分、各支血管受累情况、支架所在血管及其受累情况）；

冠状动脉造影：记录患者入院时冠状动脉造影（包括检查时间、各支血管受累情况、支架所在血管及其受累情况）；

超声心动图：包括心脏结构及功能相关参数（射血分数、左房大小、室间隔厚度、左室舒张末期及收缩末期内径）。

**5.2数据管理**

5.2.1数据录入与管理

该研究所产生的数据采用电子数据采集系统（EDC）进行录入及保存，EDC只对名单中列出的人员授权，使用个人帐号和密码登录基于互联网的数据管理系统。管理和操作EDC重要信息的工作人员需要与研究组签订保密协议。EDC管理工作人员不得擅自修改或删除现有信息。未授权人员不能访问EDC系统。

5.2.2数据质控

数据管理员每天会逐例检查所有上传的数据，若有疑问，将及时提出并提醒研究者。数据管理员由经验丰富的数据管理人员担任。所有遇到的问题都将被详细记录，包括问题内容、提问者、提出日期和解决日期。

5.2.3数据保留

所有研究记录应按照临床研究相关规定和监管机构的要求，保存在安全可靠的设施中，保留至少15年。

5.2.4研究中心的启动/培训

本研究计划中所有参与研究的人员必须完成数据收集和报告的培训。

**5.3 统计方法**

计数数据以数字和百分比表示，并使用卡方分析进行分析。正态分布的测量数据以均值±标准差表示，并使用独立样本t检验进行分析。非正态分布的连续变量以中位数和四分位数表示，并使用曼-惠特尼U检验进行分析。计数数据以数字和百分比表示，并使用卡方分析进行分析。

以有创FFR作为金标准，采用Spearman相关系数和Bland-Altman分析法分析冠脉支架术后CT-FFR新模型与QCA的相关性，计算冠状动脉支架术后CT-FFR新模型的敏感性、特异性、准确性、阳性预测值和阴性预测值及其相应的95%置信区间（CI），计算受试者工作特征曲线（ROC）分析的曲线下面积（AUC），评估训练得到的冠状动脉支架术后CT-FFR模型效果。双尾P值<0.05被认为具有统计学意义。

**6. 伦理和传播**

**6.1 研究伦理批准**

研究方案将由首都医科大学附属北京安贞医院伦理委员会、中国医学科学院阜外医院伦理委员会、首都医科大学附属北京同仁医院伦理委员会、北京医院伦理委员会、首都医科大学附属北京朝阳医院伦理委员会、首都医科大学宣武医院伦理委员会伦理委员会审核并批准。本研究方案的设计、实施和报告应遵从ICH-GCP、适用的地方性法规和世界医学协会（WMA）赫尔辛基宣言伦理规定。

在受试者注册之前，负责监督研究的伦理委员会将审查和批准研究方案、预计入选的受试者信息以及任何后续修改。研究开始之前，主要研究者必须签署研究方案签字页，确认其同意遵照研究相关文件以及本研究方案中的说明和流程开展研究，并根据要求向研究者提供所有相关数据和记录。

**6.2方案修订**

方案修订是对研究改动的书面描述或对研究方案的正式声明，其可能会影响临床研究的实施、潜在获益或者受试者安全，包括研究目的、研究的设计、研究对象、样本量、研究程序或者重要的研究管理方案。研究方案管理信息的变更对临床研究的实施方式没有重大影响，且不影响受试者的安全，属于微小的修正或澄清（例如电话号码变更，组织安排变更）。研究方案修订必须经主要研究者、监管机构（如有必要）和伦理委员会批准。如果出于保证受试者安全的目的，研究方案修订可在伦理委员会批准前实施修改。尽管研究方案的修改需要经过正式的审批流程，但为确保本研究受试者的安全，研究人员可采取相应紧急处理措施，无论该措施是否与原方案相悖。如出现此类情况，应及时通知研究中心的IRB/EC。

**6.3 知情同意**

本研究于患者招募前已取得知情同意。

**6.4 保密**

本研究实施过程中将采取一系列措施保护受试者的隐私。受试者数据在录入数据库前将去除可识别受试者身份的信息，以保护其隐私。在监测数据质量和研究方案依从性的过程中，监查人员将查阅临床/中心的医疗记录。该信息将被记录在患者信息表中。报告研究数据和结果时，所有个人及临床研究中心的信息都将被隐藏，以保护受试者的个人隐私。

**6.5 利益声明**

整个研究的所有研究者之间不存在财务和其他利益冲突。

**6.6 数据访问**

本研究的数据集经脱敏后存储于安贞医院心血管智慧诊疗中心服务器，所有数据集都将受到密码保护。

**6.7辅助治疗和研究结束后患者管理**

本研究为诊断性研究，不会对患者产生伤害。

**6.8发表策略**

6.8.1

这项研究的主要结果将以本研究的名义发表。文章撰写由指导委员会（SC）批准的编写委员会完成。

编写委员会将由各委员会成员、统计师、研究员和研究者组成。他们将以本研究的名义编写研究的主要报告。研究结果将在杂志发表，并在心血管领域的国家和国际会议上报告。

6.8.2

出版物的作者必须符合国际医学期刊编辑委员会（ICMJE）的指导方针，该规定如下：

a.作者必须对研究的概念和设计、数据的获得或数据的分析和结果的解读作出重大贡献；

b.作者必须起草出版物，或在草稿审查期间有贡献（数据分析、解读或其他重要的内容），经其他作者同意，对稿件进行重大修改；

c.在文章投稿前，作者必须批准最终版文稿。

d.只能在主文章发表后，才能发表其他研究的结果。

6.8.3

计划允许公众访问完整研究方案、参与级数据集和统计代码：无。
