# Supplementary material for: Rationale and design of a multicenter, prospective, diagnostic clinical study: A study protocol for evaluating the diagnostic validation of deep learning-based noninvasive CT-FFR for in-stent restenosis
Source: PLoS One. 2026 May 6;21(5):e0346723. doi: 10.1371/journal.pone.0346723 (PMC13148680; doi:10.1371/journal.pone.0346723)
Supplement: S3 File — (DOCX) [file pone.0346723.s003.docx]

**Deep Learning-Based Noninvasive Flow Reserve Fraction (CT-FFR) for In-Stent Restenosis Assessment**

Version: V1.0
Date: December 08, 2021

Confidential – For research use only

**Principal Investigator Statement**

I have read the following study protocol:
Deep Learning-Based Noninvasive Flow Reserve Fraction (CT-FFR) for In-Stent Restenosis Assessment
Version: 1.0
Date: December 08, 2021

I confirm that this protocol contains all necessary details to conduct the study. I will carry out the study as described and complete it within the planned timeframe. I will provide all investigators involved in this study with copies of the protocol and relevant information and ensure that they fully understand the study interventions and organizational arrangements.

**Principal Investigator**

Prof. Dongfeng Zhang
National Clinical Medical Research Center for Cardiovascular Diseases
Beijing Anzhen Hospital, Capital Medical University

Date: _____________
Signature: _____________

**Introduction (Background and Rationale)**

With rapid socioeconomic development, profound lifestyle changes, population aging, and the increasing prevalence of cardiovascular risk factors, the incidence of coronary artery disease (CAD) has been rising steadily. Percutaneous coronary intervention (PCI) has become one of the primary treatment modalities for CAD. Registry data indicate that between 2009 and 2019, the number of PCI procedures performed in mainland China increased annually. Despite the impact of the COVID-19 pandemic in 2020, the total number of PCI procedures still approached one million.

In-stent restenosis (ISR) is defined as ≥50% luminal narrowing within the stented segment or within 5 mm of the stent edge, as confirmed by invasive coronary angiography (ICA). In the era of bare-metal stents, ISR occurred in 20–40% of cases. Although improvements in stent design and the application of drug-eluting stents (DES) have markedly reduced ISR incidence, the rate remains 5–10%. ISR is strongly associated with major adverse cardiovascular events (MACE). Given the large and continuously expanding population undergoing PCI, ISR remains a major clinical challenge both now and in the future.

ICA is the gold standard for diagnosing ISR, but its invasive nature limits patient acceptance and carries potential complications, including allergic reactions to contrast agents, guidewire entanglement, vascular occlusion, bleeding, hematoma, and arteriovenous fistula. Therefore, ICA is unsuitable as a first-line screening tool for ISR. Coronary computed tomography angiography (CCTA) is widely used as a non-invasive screening method in clinical practice. However, the diagnostic accuracy of CCTA in ISR is affected by stent characteristics (diameter, thickness, and material), the degree of vascular calcification, and the location of the ISR lesion.

Fractional flow reserve (FFR) is an established invasive physiological index for assessing the hemodynamic significance of coronary stenosis. By evaluating the change in blood flow across a stenosis, FFR determines the presence and severity of myocardial ischemia, thus helping to optimize treatment and avoid overtreatment. Computational fluid dynamics (CFD), a discipline combining computer technology and fluid mechanics, has been applied to simulate blood flow in vessels and airflow/particle dynamics in the airway. By integrating CFD with CCTA, hemodynamic parameters within coronary arteries can be estimated, allowing FFR values to be calculated across the entire coronary tree. In recent years, such CCTA-based FFR techniques have become effective non-invasive methods for functional evaluation of coronary artery disease.

The DEEPVESSEL FFR system, independently developed in China, integrates artificial intelligence, medical imaging, and biomedical engineering technologies. Employing self-developed sequence-based deep learning algorithms, it enables intelligent, accurate assessment of vascular diseases within the circulatory system. It provides rapid, effective, and non-invasive calculation of FFR to assess myocardial ischemia, achieving precise, efficient detection at a lower cost.

This study aims to establish an AI-based CT-FFR model specifically for ISR, to determine the diagnostic accuracy of this new model, and to evaluate its feasibility in guiding treatment strategies. By providing precise, non-invasive, and efficient ISR assessment, this approach is expected to enable individualized treatment, reduce misdiagnosis and overtreatment, improve patient prognosis following stent implantation, enhance the overall management of CAD in Beijing and across China, and promote the construction of graded diagnosis and treatment system.

**2. Management Information**

**2.1 Trial/Study Registration**

This study protocol has been reviewed and approved by the Ethics Committee of Beijing Anzhen Hospital, Capital Medical University, as well as the ethics committees of 6 subcenters (KS2022005), and registered with the Chinese Clinical Trial Registry (ChiCTR2200058822).

**2.2 Supporting Institution**

Keya Medical serves as the collaborating institution for this study. Its primary responsibilities include data analysis, assisting in the interpretation of study results, and providing support for manuscript preparation, patent applications, and related tasks.

**2.3 Investigators and Responsibilities**

**2.3.1 Participating Centers and Principal Investigators**

Main Center: Beijing Anzhen Hospital, Capital Medical University

Principal Investigators: Xiantao Song, Dongfeng Zhang, Zhao Ma, Meichen Sun, Yifei Nie, Shiqi Liu

Subcenter: Xuanwu Hospital, Capital Medical University

Principal Investigators: Jing Li, Keling Xiao

Subcenter: Beijing Hospital

Principal Investigators: Xue Yu, Peng Li

Subcenter: Beijing Tongren Hospital, Capital Medical University

Principal Investigators: Caixia Guo, Xue Jiang

Subcenter: Beijing Chaoyang Hospital, Capital Medical University

Principal Investigators: Li Xu, Chuang Li

Subcenter: Fuwai Hospital, Chinese Academy of Medical Sciences

Principal Investigator: Jie Qian

**2.3.2 Contact Information**
Prof. Dongfeng Zhang
Email: dongfengdoctor@outlook.com

**2.3.3**
This is an investigator-initiated clinical study. All major scientific decisions and study management will be overseen by the Steering Committee.

**2.3.4 Project Management**
Project management and operational coordination will be conducted by the sponsor or a contract research organization (CRO) designated by the sponsor. Responsibilities include, but are not limited to: project and data management, coordination among members of various committees, assisting investigators in ethics committee submissions and progress reports, supporting the drafting and refinement of study protocols, training of study personnel, study center initiation, monitoring and auditing, oversight of data quality and security, ensuring compliance with the protocol, clinical guidelines, and applicable regulations, and supporting the preparation of study data and manuscripts for publication.

A Steering Committee (SC) will be established, comprising experts from the main center and subcenters. The SC is responsible for approving the final study protocol and making key decisions during study conduct, data analysis, and reporting. The SC reserves the right to add new members to strengthen study execution and analysis. Members from Keya Medical will also participate in the SC.

The Data Management Team will be responsible for maintaining the IT systems, data entry, and data validation. Data collection and management will be carried out using an Electronic Data Capture (EDC) system. The team will design the electronic case report forms (eCRF), maintain the electronic medical records, and monitor data quality throughout the study implementation.

**2.4 Glossary of Abbreviations and Terms**

CI: Confidence Interval

SD: Standard Deviation

CRF/eCRF: Case Report Form / Electronic Case Report Form

DSMB: Data and Safety Monitoring Board

EC: Ethics Committee

EDC: Electronic Data Capture

GCP: Good Clinical Practice

CRO: Contract Research Organization

IC: Informed Consent

ICMJE: International Committee of Medical Journal Editors

PI: Principal Investigator

SC: Steering Committee

**2.5 Study Synopsis**

| Title | Deep Learning-Based Noninvasive Flow Reserve Fraction (CT-FFR) for In-Stent Restenosis Assessment |
| --- | --- |
| Objectives | To establish an AI–based CT-FFR model specifically designed for the evaluation of in-stent restenosis (ISR), to determine its diagnostic accuracy, and to explore its feasibility in guiding clinical treatment strategies. |
| Study design | This is a multicenter, prospective diagnostic clinical study. |
| Reference standard | Invasive fractional flow reserve (FFR). |
| Planned Sample Size | 331 patients |
| Study Duration | 48 months |
| Study content | 250 patients from Beijing Anzhen Hospital will be used for supplementary training of the existing DEEPVESSEL FFR model, and 81 patients from the other 6 subcenters will be used in cross-validation using invasive FFR as reference standard. |
| Inclusion criteria | Prior percutaneous coronary intervention (PCI) with stent implantation.  Underwent CCTA within 3 months before invasive coronary angiography (ICA) with FFR measurement. |
| Exclusion criteria | Acute coronary syndrome (ACS) within the previous 3 months.  Prior coronary artery bypass grafting (CABG).  Severe coronary calcification, defined as coronary artery calcium score (CACS) ≥400.  Presence of significant comorbidities, including heart failure, severe valvular disease, significant arrhythmias, or other metallic implants interfering with imaging.  Determined by the investigator to be otherwise unsuitable for study participation. |
| Elimination criteria | Poor-quality CCTA images preclude CT-FFR computation. |
| Statistics | Using invasive FFR as the reference standard, sensitivity, specificity, accuracy, positive predictive value, and negative predictive value with their corresponding 95% confidence intervals (CIs) were calculated for CT-FFR. The receiver operating characteristic (ROC) curve was analyzed, and the area under the curve (AUC) was calculated. The McNemar test and Bland-Altman plot will be used to examine the diagnostic consistency between CT-FFR and invasive FFR. The correlation was analyzed by Pearson’s correlation coefficient and Spearman’s correlation coefficient. All tests were two-tailed with p<0.05 significant. |
| Data management | Study data will be collected and managed using an electronic case report form (eCRF) system. |

**3.Study content**

**3.1 Study objectives**

To establish an AI–based CT-FFR model specifically designed for the evaluation of in-stent restenosis (ISR), to determine its diagnostic accuracy, and to explore its feasibility in guiding clinical treatment strategies.

**3.2 Study design**

This study is a multicenter, prospective diagnostic clinical study.

This study will be carried out in Beijing Anzhen Hospital and 6 subcenters (Fuwai Hospital, Chinese Academy of Medical Sciences, Beijing Tongren Hospital, Beijing Hospital, Beijing Chaoyang Hospital, Xuanwu Hospital) in China. We plan to initiate prospective enrollment of 331 post–stent implantation patients who underwent coronary angiography with available CCTA and invasive FFR data (within 3 months) starting in June 2022. Among them, 250 patients from Beijing Anzhen Hospital will be used for supplementary training of the existing DEEPVESSEL FFR model, and 81 patients from the other 6 subcenters will be used in external validation.

**4. Study methods**

**4.1 Study Center Selection**
This study was initiated and is led by Beijing Anzhen Hospital, Capital Medical University. Prospective patient enrollment and data collection will begin in June 2022. We will prospectively enroll eligible patients meeting the inclusion and exclusion criteria at Beijing Anzhen Hospital and 6 subcenters (Fuwai Hospital, Chinese Academy of Medical Sciences, Beijing Tongren Hospital, Beijing Hospital, Beijing Chaoyang Hospital, Xuanwu Hospital).

**4.2 Inclusion and exclusion Criteria**

**4.2.1 Inclusion Criteria**

Patients will be eligible if they meet all of the following:

1. Prior percutaneous coronary intervention (PCI) with stent implantation.
2. Underwent CCTA within 3 months prior to invasive coronary angiography (ICA) with FFR measurement.

**4.2.2 Exclusion Criteria**

Patients meeting any of the following will be excluded:

1. Acute coronary syndrome (ACS) within the previous 3 months.
2. Prior coronary artery bypass grafting (CABG).
3. Severe coronary calcification, defined as coronary artery calcium score (CACS) ≥400.
4. Presence of significant comorbidities, including heart failure, severe valvular disease, significant arrhythmias, or other metallic implants interfering with imaging.
5. Determined by the investigator to be otherwise unsuitable for study participation.

**4.2.3 Elimination criteria**

Poor-quality CCTA images preclude CT-FFR computation.

**4.3 CT-FFR Modeling**

We will employ a Deep Bidirectional Long-term Recurrent Neural Network (DBL-RNN) algorithm, independently developed by Keya Medical, which integrates a Multi-Layer Perceptron (MLP) and a Bidirectional Recurrent Neural Network (Bi-RNN). The framework will consist of two core components: CCTA image processing and deep learning–based CT-FFR calculation.

In the CCTA image processing and feature extraction stage, a Multi-Layer Perceptron (MLP) will be utilized for fully automated three-dimensional coronary artery reconstruction, including centerline extraction and lumen segmentation. Based on the reconstructed coronary geometry, a series of morphological and hemodynamics-related local feature vectors will be extracted point-by-point along the vessel centerline. These feature vectors include local vascular features (e.g., coronary cross-sectional area, coronary radius, distance to the nearest upstream coronary bifurcation), local stenotic features (e.g., stenosis length and the smallest 50% coronary radius along the stenosis), and global features (including upstream and downstream vascular characteristics and stenosis features).

The deep learning component adopts a Bidirectional Recurrent Neural Network (Bi-RNN) architecture. The sequential feature vectors generated from the previous step will be input into the Bi-RNN, which enables bidirectional information propagation and simultaneously accounts for the local upstream and downstream context of each centerline point as well as its global relationship within the entire coronary tree. Through this architecture, the network will infer continuous CT-FFR values along the entire coronary artery tree.

**4.4 Samplesize calculation**

The sample size calculation was based on previous studies and the following hypothesis: At the patient level, CT-FFR demonstrated an AUC of 0.73(95% CI: 0.55-0.87) for detecting ISR, with a disease prevalence of 76%. A sample size calculation was performed using PASS 2021 (Test for One ROC Curve) with α = 0.05, power = 0.9, and two-tailed testing, yielding a required sample size of 77 patients. We planned to prospectively enroll 81 patients in the external validation cohort, which was expected to provide 90% power to detect an AUC of 0.73.

The training cohort size was defined based on data availability and a predefined training-to-validation ratio of approximately 3:1 to balance model development and independent performance evaluation. Therefore, at least 243 patients were required for model training. Considering clinical practice and data availability, we prospectively planned to enroll 250 patients to meet the sample size requirement, which is comparable to or even larger than the training cohort sizes reported in previous study.

**5. Data collection, management and analysis**

**5.1 Data collection**

**5.1.1 Demographic Data**

Age, Sex, Height, Weight and Dates of hospital admission and discharge.

**5.1.2 Medical History**

Chief complaint at admission, Present illness, Past medical history (including hypertension, diabetes mellitus, dyslipidemia, previous myocardial infarction, and disease duration), Personal history (smoking, alcohol consumption), Medication history (prior use of cardiovascular or related drugs) and Family history (cardiovascular diseases), Previous PCI procedure date(s).

**5.1.3 Laboratory Tests**

Complete blood count, C-reactive protein (CRP), High-sensitivity troponin I/T (hs-TnI/TnT), Creatine kinase-MB (CK-MB), B-type natriuretic peptide (BNP), N-terminal pro-B-type natriuretic peptide (NT-proBNP), D-dimer, Fibrin degradation products (FDP), Liver and renal function tests, Lipid profile, Blood glucose, and other relevant biochemical markers.

**5.1.4 Clinical Examinations**

Vital signs and physical examination: blood pressure and heart rate at admission

Electrocardiogram (ECG): ECG findings at admission

Coronary CTA: examination details at admission, including scan date, calcium score, extent of vessel involvement, and stent location and involvement

Coronary angiography (ICA): angiographic findings at admission, including procedure date, extent of vessel involvement, and stent location and involvement

Echocardiography: cardiac structural and functional parameters, including left ventricular ejection fraction (LVEF), left atrial diameter, interventricular septal thickness, and left ventricular end-diastolic and end-systolic diameters

**5.2.1 Data Entry and Management**

Study data will be entered and stored using an electronic data capture (EDC) system. Access to the EDC system will be granted only to authorized personnel listed in the study roster. Each authorized user will log into the web-based data management system with an individual account and password. Personnel responsible for managing and operating critical EDC functions are required to sign a confidentiality agreement with the study group. EDC managers are not permitted to modify or delete existing information without authorization. Unauthorized individuals will have no access to the EDC system.

**5.2 Data management**

**5.2.2 Data Quality Control**

Data managers will review all uploaded data daily and flag any queries to the investigators promptly. Data managers will be experienced personnel with professional training in data management. All identified issues will be documented in detail, including the content of the query, the person raising the query, the date raised, and the date resolved.

**5.2.3 Data Retention**

All study records will be preserved in secure and reliable facilities, in accordance with applicable clinical research regulations and requirements of regulatory authorities. Data will be retained for a minimum of 15 years after study completion.

**5.2.4 Study Initiation and Training**

All study personnel must complete training on data collection and reporting procedures prior to study initiation.

**5.3 Statistical Methods**

Categorical variables will be expressed as counts and percentages, and analyzed using the chi-square test. Normally distributed continuous variables will be presented as mean ± standard deviation (SD) and compared using the independent-samples t-test. Non-normally distributed continuous variables will be expressed as median with interquartile range (IQR) and analyzed using the Mann–Whitney U test.

Using invasive FFR as the reference standard, sensitivity, specificity, accuracy, positive predictive value, and negative predictive value with their corresponding 95% confidence intervals (CIs) were calculated for CT-FFR. The receiver operating characteristic (ROC) curve was analyzed, and the area under the curve (AUC) was calculated. The McNemar test and Bland-Altman plot will be used to examine the diagnostic consistency between CT-FFR and invasive FFR. The correlation was analyzed by Pearson’s correlation coefficient and Spearman’s correlation coefficient. All tests were two-tailed with p<0.05 significant.

**6. Ethics and Dissemination**

**6.1 Ethical Approval**

The study protocol will be reviewed and approved by the Institutional Review Boards (IRBs)/Ethics Committees of Beijing Anzhen Hospital and all subcenters.

The design, conduct, and reporting of this study will comply with the International Council for Harmonisation Good Clinical Practice (ICH-GCP) guidelines, all applicable local regulations, and the ethical principles of the World Medical Association (WMA) Declaration of Helsinki.

Before the enrolment of any participants, the responsible ethics committees will review and approve the study protocol, information regarding the anticipated study population, and any subsequent amendments. Before study initiation, the principal investigator must sign the protocol signature page to confirm agreement to conduct the study in accordance with the protocol, associated documents, and specified procedures, and to provide all required data and records to the investigators as requested.

**6.2 Protocol Amendments**

A protocol amendment is defined as a written description of changes to the study or a formal statement of modifications to the protocol that may affect study implementation, potential benefits, or participant safety. Such changes may involve study objectives, design, target population, sample size, procedures, or important aspects of study management.

Minor administrative changes that do not significantly impact study conduct or participant safety (e.g., change of telephone numbers, organizational arrangements) will be considered administrative modifications or clarifications.

All protocol amendments must be approved by the principal investigator, regulatory authorities (if applicable), and the responsible Ethics Committees. Amendments undertaken to ensure participant safety may be implemented prior to Ethics Committee approval if necessary. Although protocol modifications must undergo a formal approval process, investigators may implement urgent measures to protect study participants, regardless of whether such measures deviate from the original protocol. In such cases, the relevant Institutional Review Board (IRB)/Ethics Committee must be notified promptly.

**6.3 Informed Consent**

Informed consent was obtained from all participants prior to patient enrollment.

**6.4 Confidentiality**

Strict measures will be taken to protect participant privacy throughout the study. All identifying information will be removed from study data prior to entry into the database to ensure confidentiality. During monitoring of data quality and protocol compliance, monitors may review clinical or institutional medical records. Such information will be documented in the patient information form. When study data and results are reported, all identifying details of participants and study centers will be concealed to maintain confidentiality.

**6.5 Declaration of Interests**

The investigators declare no financial or other conflicts of interest related to this study.

**6.6 Data Access**

The de-identified study dataset will be stored on secure servers of the Cardiovascular Intelligent Medicine Center of Beijing Anzhen Hospital. All datasets will be password-protected and accessible only to authorized personnel.

**6.7 Adjunctive Therapy and Post-Study Patient Management**

As this is a diagnostic study, it does not impose any interventions on patients and will not cause harm.

**6.8 Publication Policy**

**6.8.1 Primary Publication**

The main results of this study will be published under the name of the study. Manuscript preparation will be carried out by a writing committee approved by the Steering Committee (SC). The writing committee will consist of SC members, statisticians, fellows, and investigators. They will prepare the primary report of the study on behalf of the study group. Study results will be submitted for publication in peer-reviewed journals and presented at national and international cardiovascular conferences.

**6.8.2 Authorship**

Authorship of publications must comply with the guidelines of the International Committee of Medical Journal Editors (ICMJE), which require the following:
a. Authors must make a substantial contribution to the conception and design of the study, the acquisition of data, or the analysis and interpretation of results.
b. Authors must draft the publication or contribute substantially during manuscript revision (e.g., through data analysis, interpretation, or other critical content), and must participate in important revisions approved by all co-authors.
c. Authors must approve the final version of the manuscript before submission.
d. Secondary publications of study findings may only be submitted after the main article has been published.

**6.8.3 Data Sharing Statement**

Plans for public access to the full study protocol, participant-level dataset, and statistical code: None.
