# Supplementary material for: Rationale and design of a multicenter, prospective, diagnostic clinical study: A study protocol for evaluating the diagnostic validation of deep learning-based noninvasive CT-FFR for in-stent restenosis
Source: PLoS One. 2026 May 6;21(5):e0346723. doi: 10.1371/journal.pone.0346723 (PMC13148680; doi:10.1371/journal.pone.0346723)
Supplement: S4 File — (DOCX) [file pone.0346723.s004.docx]

**Institutional Review Board (IRB) Approval Letter**

**Beijing Anzhen Hospital, Capital Medical University**

| Study Title | Deep Learning-Based Noninvasive Flow Reserve Fraction (CT-FFR) for In-Stent Restenosis Assessment | | |
| --- | --- | --- | --- |
| Source of Funding: | Beijing Nova Program | Researcher | Dongfeng Zhang |
| Version Number of Protocol | V1.0 | Date of Protocol | 20211208 |
| Version Number of Informed Consent | V1.0 | Date of Informed Consent | 20211208 |
| Approval number | KS2022005 | | |
| **Primary Reviewer’s Comments** | | | |
| ☑Approved □Approved with Modifications □Resubmission Required after Modifications □Termination of the Approved Study □Not Approved | | | |
|  | Signature of the Primary Reviewer: Xiantao Song | | |
| ☑Approved □Approved with Modifications □Resubmission Required after Modifications □Termination of the Approved Study □Not Approved | | | |
|  | Signature of the Primary Reviewer: Hai Gao | | |
| ☑Approved □Approved with Modifications □Resubmission Required after Modifications □Termination of the Approved Study □Not Approved | | | |
|  | Signature of the Primary Reviewer: Yang Yu | | |
| **Expedited Review Comments** | | | |
| Expedited Review Comments | Approved | | |
| Chair of the Ethics Committee | Lei Xv | Ethics Committee  (Official Seal) |  |
|  |  | Date of Approval | 2022.2.11 |
