# Supplementary material for: Rationale and design of a multicenter, prospective, diagnostic clinical study: A study protocol for evaluating the diagnostic validation of deep learning-based noninvasive CT-FFR for in-stent restenosis
Source: PLoS One. 2026 May 6;21(5):e0346723. doi: 10.1371/journal.pone.0346723 (PMC13148680; doi:10.1371/journal.pone.0346723)
Supplement: S5 File — (PDF) [file pone.0346723.s005.pdf]

首都医科大学附属北京安贞医院
医学伦理委员会
快速审查批复单

项目基本情况：

项目名称	基于深度学习的无创血流储备分数（CT-FFR）评估支架内再狭窄的研究		
项目来源	**北京市科技新星计划**	研究者	张东凤
方案版本号	V1.0	方案版本日期	20211208
知情同意书版本号	V1.0	知情同意书版本日期	20211208
伦理编号	KS2022005		

主审意见：

☑同意、☐修改后同意、☐修改后重申、☐终止已批准的研究、☐不同意<br>主审委员签字：[illegible]
☑同意、☐修改后同意、☐修改后重申、☐终止已批准的研究、☐不同意<br>主审委员签字：[illegible]
☑同意、☐修改后同意、☐修改后重申、☐终止已批准的研究、☐不同意<br>主审委员签字：[illegible]

快速审查意见：

快速审查意见	同意		
伦理主任委员<br>/副主任委员	[illegible]	伦理委员会<br>（盖章）	首都医科大学附属北京安贞医院<br>伦理委员会
伦理快速审查意见签发日期：2022年 2月 11日			
