# Supplementary material for: Rationale and design of a multicenter, prospective, diagnostic clinical study: A study protocol for evaluating the diagnostic validation of deep learning-based noninvasive CT-FFR for in-stent restenosis
Source: PLoS One. 2026 May 6;21(5):e0346723. doi: 10.1371/journal.pone.0346723 (PMC13148680; doi:10.1371/journal.pone.0346723)
Supplement: S6 File — (PDF) [file pone.0346723.s006.pdf]

基于深度学习的无创血流储备分数（CT-FFR）评估支架内再狭窄的研究

0000001

尊敬的患者:

您如愿意参加一项由首都医科大学附属北京安贞医院张东凤教授牵头的关于“基于深度学习的无创血流储备分数（CT-FFR）评估支架内再狭窄的研究”，请仔细阅读下列信息，然后决定是否参加本项研究，如有疑问，请询问您的医师。

背景和目的

冠状动脉造影是诊断支架内再狭窄（ISR）的金标准，但其为有创检查，患者依从性差，且本身有麻醉剂过敏、出血等多种并发症可能，因此不适宜临床上筛选 ISR 的首选方案。冠状动脉 CT 血管造影（CCTA）是目前实际诊疗工作中应用较为普遍的筛查方案。计算流体力学（CFD）是计算机技术和流体力学相结合的交叉学科，CFD 技术已经被应用到血液在血管里流动、气体/颗粒在肺气管里流动仿真模拟，将 CFD 与 CCTA 结合可以推导冠状动脉内血流的流体动力学参数，从而计算出整个冠脉树上的血流储备分数（FFR）值。我国自主研发的 DEEPVESSEL FFR 模型通过图像处理技术，进行大量数据分析，提取特征参数得到经过验证的训练模型，并应用到新的测量数据上，对人体循环系统疾病进行智能精准评估，快速、有效、无创的计算用于评估心肌缺血的 FFR，实现精确高效检测，并达到低成本诊断的目的。本研究旨在明确基于人工智能的精准评估支架内再狭窄的无创血流储备分数模型（CT-FFR）的诊断准确性及指导治疗的可行性。

入选/排除标准

您已被证实符合下述情况：1、年龄 18-80 岁，既往支架植入术后至少 3 个月，再发心绞痛症状的冠心病患者；2、无支架内血栓、急性心肌梗死，无严重心功能不全、肝肾功能不全、主动脉缩窄；3、没有 CCTA 扫描禁忌及未处于怀孕或育龄妇女状态。则您适合参加本项研究，因此请您考虑是否自愿参加本项研究。如果您同意参加本研究，按研究计划，需进行 CCTA、CT-FFR、冠脉造影及有创 FFR 检查。

风险与获益

风险主要为手术相关并发症，如麻醉剂及相关并发症；血管相关并发症，如局部穿刺部位出血、血肿、感染、假性动脉瘤或动静脉瘘形成等；造影剂相关并发症如造影剂过敏，造影剂肾病等；神经相关并发症；冠状动脉并发症如冠脉痉挛、夹层、撕裂、无复流及慢血流现象或急性冠脉血栓形成、闭塞，严重导致心肌缺血、心肌梗死或冠脉穿孔导致心包填塞；术中/术后心、脑血管意外事件发生；手术器械相关并发症；血管缝合器相关并发症等。通过应用 FFR 等检查，可能避免不必要的支架植入，减少相关操作带来的风险，优化您的整体治疗策略。

自愿原则

您是否参加此项研究完全出于自愿，您可以不参加此项临床研究或者在任何时间退出此项研究，而不需要说明任何原因，并且您不会因此失去任何益处或受到报复，也不会影响您接受其他治疗的权利。若在研究期间，您的主治医师发现您已不适合继续参加本研究时，为了确保您的健康和研究

知情同意书 V1.0

方案的顺利实施，他／她可以不经您同意而中断您参加此项研究。在研究期间，您需配合检查和治疗。

研究程序

如果您符合本研究入选标准，您即可参加本研究。

请仔细阅读本知情同意书，并向您的医生或研究人员提出任何您需要了解的问题，直至得到满意的答复。在得到所有问题的满意回答后，请在知情同意书上签字。

参加与退出

您参加本研究的决定是出于自愿。如果您决定不参加本研究，您的治疗质量不会受到影响。您可以在研究开始前或开始后的任何时候退出。

保密和原始文件保存

您授权使用您可确认的个人健康信息的个人或组织包括：您的医生，研究组，伦理委员会，国家的政府监管代理机构。所有数据都将匿名收集，并根据适用的法律法规，采取一切方法保护您的记录和身份的保密性。如果发表本研究的结果，将对您的身份保密。您有权获得本研究中与您有关的信息。

签署

在医生向我解释了本研究，我的问题已经得到了满意的答复；并且我知道我有权在任何时候退出本研究项目而利益不会受到损失后，我在本同意书上签名，证明我自愿同意参加本研究。并且我确认我收到了该知情同意书的复件。

知情同意书签署页

我已知晓关于基于深度学习的无创血流储备分数（CT-FFR）评估支架内再狭窄的研究介绍。我的医师已就此治疗的特点及可能存在的副作用向我作了详细解释说明，对我询问的所有问题也给予了解答。如果我不参加此次研究或中途退出，将不会影响我的医疗保障，也不会受到任何歧视和报复，而且不影响我应有的合法权利。

在此，我自愿参加本项研究。

受试者签名: [illegible] 日期: 2022-6-15

*受试者亲属签名: [illegible] 与受试者关系: [illegible] 日期: 2022-6-15

地址: [illegible] 联系电话: [redacted]

（*指受试者为无签字能力者）

我或我的研究人员已向该受试者充分解释和说明了本临床试验的目的、操作过程以及受试者参加该试验可能存在的风险和潜在的利益，并满意地回答了受试者的所有有关问题。

主要研究者或研究者指定的研究人员（对受试者进行告知者）签名: 张东凤

日期: 2022-6-15 联系电话: [redacted]
