# Supplementary material for: Rationale and design of a multicenter, prospective, diagnostic clinical study: A study protocol for evaluating the diagnostic validation of deep learning-based noninvasive CT-FFR for in-stent restenosis
Source: PLoS One. 2026 May 6;21(5):e0346723. doi: 10.1371/journal.pone.0346723 (PMC13148680; doi:10.1371/journal.pone.0346723)
Supplement: S7 File — (DOCX) [file pone.0346723.s007.docx]

**A Study for Evaluating the Diagnostic Validation of Deep Learning-Based Noninvasive CT-FFR for In-Stent Restenosis**

**No. 0000001**

**Dear Patient,**

You are invited to participate in a research study led by Professor Dongfeng Zhang at Beijing Anzhen Hospital, Capital Medical University, entitled **“A Study for Evaluating the Diagnostic Validation of Deep Learning-Based Noninvasive CT-FFR for In-Stent Restenosis.”**

Please read the following information carefully and decide whether you wish to participate in this study. If you have any questions, please consult your physician before making your decision.

**Background and Purpose**

Coronary angiography is considered the gold standard for diagnosing in-stent restenosis (ISR). However, it is an invasive procedure associated with relatively low patient acceptance and potential complications, including allergic reactions to anesthetic agents and bleeding. Therefore, it is not ideal as a first-line screening tool for ISR in routine clinical practice.

Coronary computed tomography angiography (CCTA) is currently a widely used noninvasive screening modality in clinical practice. Computational fluid dynamics (CFD), an interdisciplinary field combining computer science and fluid mechanics, has been applied to simulate blood flow within vessels and air or particle flow in airways. By integrating CFD with CCTA, hemodynamic parameters of coronary blood flow can be derived, enabling calculation of fractional flow reserve (FFR) values throughout the entire coronary artery tree.

The domestically developed DEEPVESSEL FFR model applies advanced image processing and large-scale data analysis to extract relevant features and generate validated trained models, which are then applied to new angiographic data for intelligent and precise assessment of cardiovascular diseases. This approach allows for rapid, effective, and noninvasive calculation of FFR to evaluate myocardial ischemia, achieving accurate detection with reduced cost.

The purpose of this study is to evaluate the diagnostic accuracy and clinical feasibility of an artificial intelligence–based noninvasive CT-FFR model for assessing in-stent restenosis and guiding treatment decisions.

**Inclusion and Exclusion Criteria**

You have been determined to meet the following criteria:

1. Age between 18 and 80 years, with a history of coronary stent implantation within the last 3 months, and recurrent angina symptoms;
2. No in-stent thrombosis or acute myocardial infarction; no severe heart failure, hepatic or renal dysfunction, or aortic coarctation;
3. No contraindications to CCTA scanning and not currently pregnant or of childbearing status.

Based on the above, you are eligible to participate in this study. If you agree to participate, the study protocol requires that you undergo CCTA, CT-FFR analysis, coronary angiography, and invasive FFR measurement.

**Risks and Benefits**

Potential risks are mainly related to procedural complications, including but not limited to:

1. Anesthesia-related adverse reactions;
2. Vascular complications such as bleeding, hematoma, infection, pseudoaneurysm, or arteriovenous fistula at the puncture site;
3. Contrast-related complications such as allergic reactions or contrast-induced nephropathy;
4. Neurological complications;
5. Coronary complications such as coronary spasm, dissection, tearing, no-reflow or slow-flow phenomena, acute coronary thrombosis or occlusion, which may result in myocardial ischemia, myocardial infarction, or pericardial tamponade due to coronary perforation;
6. Perioperative or postoperative cardiovascular or cerebrovascular events;
7. Device-related complications, including those related to vascular closure devices.

By applying physiological assessments such as FFR, unnecessary stent implantation may be avoided, procedural risks may be reduced, and your overall treatment strategy may be optimized.

**Voluntary Participation**

Your participation in this study is entirely voluntary. You may choose not to participate or to withdraw from the study at any time without providing any reason. Your decision will not result in any loss of benefits, retaliation, or negative impact on your right to receive other medical treatments.

During this study, if your treating physician determines that continued participation is no longer appropriate, participation may be discontinued without your consent to ensure your safety and the integrity of the study. During the study period, you are expected to cooperate with the required examinations and treatments.

**Study Procedures**

If you meet the inclusion criteria, you may participate in this study.

Please read this informed consent form carefully and ask your physician or study personnel any questions you may have until you are fully satisfied with the answers. After all your questions have been adequately addressed, please sign this informed consent.

**Participation and Withdrawal**

Your decision to participate in this study is voluntary. If you decide not to participate or to withdraw at any time before or after the study begins, the quality of your medical care will not be affected.

**Confidentiality and Data Retention**

You authorize the use of your identifiable personal health information by the following individuals or organizations: your physicians, the research team, the ethics committee, and relevant governmental regulatory authorities.

All data will be collected in an anonymized manner, and all reasonable measures will be taken to protect the confidentiality of your records and identity in accordance with applicable laws and regulations. If the study results are published, your identity will remain confidential. You have the right to access information related to you that is generated during the study.

**Statement of Consent**

I confirm that the study has been explained to me by the physician, that all my questions have been answered satisfactorily, and that I understand I have the right to withdraw from the study at any time without any loss of benefits. By signing this form, I voluntarily agree to participate in this study and confirm that I have received a copy of this informed consent form.

**Informed Consent Signature Page**

I have been informed about the study entitled **“A Study for Evaluating the Diagnostic Validation of Deep Learning-Based Noninvasive CT-FFR for In-Stent Restenosis.”** My physician has explained the characteristics of this study and its potential risks and side effects in detail, and all my questions have been answered.

I understand that refusal to participate or withdrawal from this study will not affect my medical coverage, will not result in discrimination or retaliation, and will not compromise my legal rights.

I voluntarily agree to participate in this study.

Participant’s Signature: ____________________  Date: __________

Signature of Participant’s Family Member: ____________________

Relationship to Participant: ____________________  Date: __________

Address: ____________________  Contact Number: ____________________

I and our members of the research team have fully explained the purpose, procedures, potential risks, and benefits of this clinical study to the participant and have satisfactorily answered all related questions.

Signature of Principal Investigator or Authorized Researcher: ___________________

Date: __________  Contact Number: ____________________
